# Heterogeneous Catalysis Is Full of Challenges

**DOI:** 10.3390/molecules31071213

**Published:** 2026-04-07

**Authors:** Lin Huang, Yinghuai Zhu

**Affiliations:** 1 Institute of Sustainability for Chemicals, Energy and Environment, Agency for Science, Technology and Research, 1 Pesek Road, Jurong Island, Singapore 627833, Singapore; 2 School of Pharmacy, Macau University of Science and Technology, Avenida Wai Long, Taipa, Macau 999078, China

Heterogeneous catalysis is a vibrant branch of chemistry, in that it enables the fast, selective and large-scale production of chemicals. Approximately 35% of the world’s GDP is influenced by catalysis [1]. As much as 80% of catalytic processes relate to heterogeneous catalysis [2]. And the production of 90% of chemicals by volume is assisted by heterogeneous catalysts [3].

The history of heterogeneous catalysis (excluding heterogeneous biocatalysis) originates from prior to the 19th century [4]. The impact of heterogeneous catalysts on the world is illustrated through the following brief overview of its key historical developments and applications.

In 1736, the first large-scale venture in H_2_SO_4_ manufacture by Joshua Ward marked the beginning of the considerable impact heterogeneous catalysis would have on the industry [5]. The process which Ward utilized involved burning sulfur with NaNO_3_ to produce H_2_SO_4_ in 60-gallon glass globes. This successful venture was succeeded by John Roebuck’s effective industrialization of H_2_SO_4_ production by burning sulfur with KNO_3_ in lead-lined chambers in 1746 [5,6]. The latter process remained the standard for H_2_SO_4_ production for almost two centuries, with a purity of 62% and a conversion of 75%. In 1817–1820, Humphry Davy discovered that platinum wire acts as a catalyst in the oxidation of coal gas, and Edmund Davy observed that finely divided platinum catalyzes reactions at room temperature [4,7]. Their work paved the way for modern industrial oxidation processes. In 1868, Henry Deacon invented a process for Cl_2_ production from the oxidation of HCl over CuCl_2_ at 350–500 °C [8]. The produced Cl_2_ was used to manufacture commercially available bleaching powder, and at the same time, the emission of HCl was curtailed. At a later date, great progress in heterogeneous catalysis was made by Paul Sabatier, who pioneered catalytic hydrogenation in 1897, discovering that finely divided metals, particularly nickel, facilitate the addition of H_2_ to unsaturated organic compounds [9,10]. This method revolutionized industrial chemistry, enabling the production of margarine, methanol and various hardened fats. At the beginning of the 20th century, the Sabatier process, involving the hydrogenation of CO_2_ to CH_4_ and water over a nickel catalyst at 300–400 °C and 30 bar, was born [9]. This reaction is currently adopted as a means to produce water onboard the International Space Station [9,10]. Sabatier was awarded the 1912 Nobel Prize in chemistry for his achievements in the hydrogenation of organic compounds using finely divided metals, along with Victor Grignard, which marked the first Nobel Prize recognition of outstanding contributions to heterogeneous catalysis.

With the development of the chemical industry in Europe and America, heterogeneous catalysis has been playing a vital role in satisfying increasing demand for bulk chemicals since the end of the 19th century. A typical example concerns the manufacture of NH_3_ from N_2_ and H_2_ by the Haber–Bosch process using iron-based catalysts, which was developed in 1910 [11]. This process enabled a dramatic increase in agricultural production using synthetic fertilizers. Frize Haber won the 1918 Nobel Prize in chemistry for developing the Haber–Bosch process, and Carl Bosch shared the 1931 Nobel Prize in chemistry for his role in this high-pressure process with Friedrich Bergius. Another renowned example involves the synthesis of polymers of olefins using Ziegler–Natta catalysts (i.e., titanium-based coordination catalyst systems). Ziegler–Natta catalysts were discovered for the polymerization of ethylene and propylene at low pressures in 1953–1954 [12], and have been used in the commercial manufacture of various polyolefins since 1956 [13]. Plastics, elastomers and rubbers produced from olefins using Ziegler–Natta and related catalysts currently represent the largest-volume commodity chemicals in the world. For their contributions to the synthesis of polymers, Karl Ziegler and Giulio Natta were jointly awarded the 1963 Nobel Prize in chemistry. Apart from the world’s economy, heterogeneous catalysis significantly impacts environmental protection. A well-known example relates to the development of catalytic converters for automobiles after the end of the 19th century [14]. Catalytic converters use supported precious metals like platinum, palladium and rhodium as catalysts to convert toxic engine gases such as CO and NO_x_ into less harmful substances such as CO_2_, N_2_ and water vapor, reducing air pollution.

The understanding of, and theories concerning, heterogeneous catalysis have progressively been developed further with the increase in and expansion of its practice since its inception. In 1823, Johann Döbereiner observed that a platinum sponge caused a jet of H_2_ to spontaneously ignite in air at room temperature without being consumed [4,15]. He developed a commercial lighter based on this principle. More importantly, he realized that the platinum sponge could lower the activation energy required for the H_2_-O_2_ reaction, allowing it to occur at room temperature [15,16]. By lending energy, the platinum sponge could adsorb H_2_ to form intermediate compounds and thus to provide an alternative reaction pathway. This effect ignited the concept of catalysis, and in 1835, the term “catalysis” was coined by Jacob Berzelius [15,16]. In 1911, Sabatier proposed a qualitative chemical theory for heterogeneous catalysis, i.e., the Sabatier principle, that an ideal catalyst binds reactants with intermediate strength (neither too strong nor too weak) [10,17]. It was further developed into “volcan plots” in the 2000s [10,18]. At present, the Sabatier principle remains fundamental in heterogeneous catalysis and electrocatalysis for identifying efficient materials such as specific metals for H_2_ evolution and CO_2_ reduction. In 1916, Irving Langmuir introduced the theory of chemisorption, moving towards a quantitative, molecular-level understanding of adsorption [19,20]. The theory models monolayer adsorption of gas molecules onto solid surfaces, assuming equivalent, distinct sits, and no lateral interaction between adsorption and desorption. Its significance lies in quantifying adsorption processes, determining maximum adsorption and providing a basis for understanding surface chemistry. For his pioneering investigations in surface chemistry, Langmuir was awarded the 1932 Nobel Prize in chemistry. In 1964–1965, Pierre Hohenberg and Walter Kohn formally established density-functional theory (DFT) based on the 1927 Thomas–Fermi model, which made DFT a standard tool for modeling complex systems [21]. The development of Generalized Gradient Approximation functionals in the 1980s improved accuracy, making DFT applicable to real-world chemical systems. Kohn’s achievements in developing DFT earned him the 1998 Nobel Prize in chemistry, along with John Pople. Since the 1960s, advanced surface techniques have allowed scientists to study catalysis at the atomic level. Advanced techniques like LEED, AES and STM were used to study surfaces and thus provide modern, atomic-level insights into surface chemistry. Gerhard Ertl and Gábor Somorjai pioneered research on the molecular-level structure, bonding, and reactivity of surfaces, particularly in catalysis, bridging the gap between surface science and industrial applications [22,23]. In 2007, the Nobel Prize in chemistry was awarded to Ertl in recognition of his contributions to modern surface chemistry and a deeper understanding of heterogeneous catalysis.

Today, heterogeneous catalysis remains essential for green chemistry, sustainability and environmental protection all over the world.

The impact of heterogeneous catalysis on the world is notably reflected in the Nobel Prize recognition. It is proudly noted that, between 1901 and 2025, the Nobel Foundation recognized outstanding achievements related to heterogeneous catalysis or catalysts 17 times, accounting for 15% of the 117 Nobel Prizes in chemistry [24]. These 17 prizes were awarded to the laureates in 1909, 1912, 1918, 1931, 1932, 1950, 1963, 1994, 1996, 1998, 2000, 2001, 2005, 2007, 2010, 2019 and 2025.

Heterogeneous catalysis encompasses a broad range of catalyzing solids and highly relevant industrial processes for the production of materials, fine chemicals and fuels [25]. Subjects of academic and industrial research in this field span from the atomic to the macroscopic scale, from fast bond making/breaking processes to slow catalyst deactivation timescales [26]. The present Special Issue titled “Research on Heterogeneous Catalysis—2nd Edition”, with 21 contributions, is devoted to new developments in heterogeneous catalysis within a broad scope. The research presented in the Special Issue demonstrates how the design of heterogeneous catalyst systems and the exploration of new materials can lead to remarkable enhancements in activity, selectivity and stability, thereby paving the way for both value-added and sustainable chemical processes.

The design of heterogeneous catalyst systems is an art, one of working on the right catalytic active components to produce the desired chemistry in catalysis. Choice and control of catalytic components require innovative strategies and sophisticated chemistry to satisfy practical processes. Brandão et al. succeeded in the breakthrough of the catalytic hydrodeoxygenation of biomass-derived feedstocks for the synthesis of hydrocarbon biofuels adopting activated carbon-supported Pt (Pt/AC) [27]. Their precisely prepared Pt/AC was very effective and led to the complete deoxygenation of lauric acid and coconut oil, yielding products composed primarily of *n*-alkanes. Sharma et al. achieved notable progress in the catalytic oxidation of dimethyl sulfide as a volatile organic compound from gaseous emission streams using WO_3_/V_2_O_5_/TiO_2_ [28]. Their work evidenced that the catalytic active component of the WO_3_/V_2_O_5_/TiO_2_ is the V on the anatase TiO_2_, and the presence of WO_3_ stabilizes the anatase crystal structure. Evidently, the incorporation of WO_3_ onto an anatase TiO_2_ support can widen the temperature range at which the WO_3_/V_2_O_5_/TiO_2_ maintains the anatase crystal structure. The WO_3_/V_2_O_5_/TiO_2_ was thus capable of effectively oxidizing dimethyl sulfide at a low temperature of 250 °C, even at an elevated dimethyl sulfide concentration of 1.6 vol % in air. Finally, Hamieh et al. developed a highly effective photo–Fenton hybrid catalyst system, FeCr-SBA-15/AC, for the degradation of methyl orange as a model high-optical-density pollutant [29]. The FeCr-SBA-15/AC achieved a methyl orange degradation efficiency of 97% under optimized conditions, which outperformed FeCr-SBA-15 by 20% due to the synergistic effect of adsorption and photo–Fenton.

It is worth emphasizing that nanomaterials have made substantial developments and have found wide applications in heterogeneous catalysis since the beginning of this century. Nanosized catalysts display higher specific activity and fewer mass transfer limitations than their microsized counterparts in heterogeneous catalytic reactions. In all, 11 of the 21 contributions in this Special Issue are concerned with heterogeneous catalysis by nanomaterials. Bikbashev et al. prepared astounding NiO nanoparticles by the solvothermal method and successfully applied them to catalytic CO_2_ methanation [30]. Under typical reaction conditions (450 °C, 30 bar, a total gas flow rate of 66 mL min^−1^ and a CO_2_/H_2_/He ratio of 1/4/6) in a fixed-bed reactor, the NiO nanoparticles could deliver a CO_2_ conversion of close to 90% and a selectivity to CH_4_ of nearly 100% with good stability within 20 h, corresponding to a rather stable turnover frequency of 118 mol CH_4_ (mol NiO)^−1^ h^−1^ for the production of CH_4_. In contrast, the NiO microparticles were obviously disadvantageous. Ding et al. made unusual nickel and copper bimetallic nanoparticles supported on HNO_3_-modified rice husk-based porous carbon (RHPC) by the impregnation method, for use in the liquid-phase hydrogenation of furfural to tetrahydrofurfuryl alcohol [31]. Under typical reaction conditions (50 °C, 1 MPa, water as solvent, 1 h and a (Ni + Cu) concentration of 20 mol %) in a batch reactor, the Ni_2_Cu_1_/RHPC, as an excellent catalyst system, gave rise to a furfural conversion of 100% and a selectivity to tetrahydrofurfuryl alcohol of 99%. This catalyst system could be recycled at least five times without loss of activity and selectivity. The exciting catalytic performance was attributed to the synergistic effect of nickel and copper bimetallic nanoparticles, on the one hand, and the promotional effect of the modified RHPC, on the other. This case broadens the application of non-noble bimetallic catalyst systems in the catalytic hydrogenation of furfural. Gajić et al. investigated the effect of polyaniline on the structure and performance of carbonized nanocomposites composed of polyaniline and TiO_2_ nanotubes, focusing on their dye adsorption capacity and photocatalytic degradation efficiency [32]. Their work hypothesized that polyaniline forms conductive carbon domains and stabilizes the anatase phase during thermal treatment, enhancing the photocatalytic performance of TiO_2_ nanotubes. Such a particular effect of polyaniline enabled TiO_2_ nanotube-based, carbon-rich nanocomposites to strongly adsorb dyes such as Acid Orange 7, Methylene Blue and Rhodamine B and effectively photocatalyze the dye degradation. Finally, dos Santos et al. provided insights into the formation of FeWO_4_ as a photocatalyst for the H_2_O_2_-assisted sunlight photocatalytic degradation of organic dyes like Methylene Blue, while investigating the photocatalytic performance of nanostructured FeWO_4_ [33]. Their work originally illustrated that the morphological control of FeWO_4_ synthesized from FeSO_4_·7H_2_O is sufficient to tune the photocatalytic performance, which distinguishes it from previously reported work, where improvements in the photocatalytic performance starting with FeWO_4_ require complex and expensive modifications of FeWO_4_, such as doping, heterostructures and dispersion. Their nanostructured FeWO_4_ could achieve the rapid and efficient degradation of Methylene Blue under sunlight with only low doses of H_2_O_2_. Their work demonstrated the potential of nanostructured FeWO_4_ as a sustainable and efficient photocatalyst for applications in water purification.

Heterogeneous catalysis is confronting multiple challenges from fine chemistry, biomass upgrading, climate change, clean energy, sustainable chemical technologies and environmental protection, due to soaring demands for hydrogen energy, fine chemicals, food, waste recycling and controlling CO_2_ emissions. Although research on heterogeneous catalysis faces many challenges, considerable advancements have been made in this field. The contributions in this Special Issue provide knowledge of chemistry and heterogeneous catalysis, multiple analytical studies, mechanistic insights and technological innovations. These achievements will contribute valuable perspectives for future challenges and orientations in heterogeneous catalysis. The global heterogeneous catalyst market size was valued at USD 21.3 billion in 2022 and is projected to reach USD 30.2 billion by 2030, growing at a CAGR of 4.5% from 2023 to 2030 [34]. This certainly creates a strongly encouraging opportunity for R&D in this field. From the research presented in this Special Issue, it is evident that genuine heterogeneous catalyst systems can render both the valorization of biomass-derived feedstocks viable [27,31,35] and the degradation of environmental pollutants efficient [28,29,30,32,33,36,37,38,39,40]. Towards the numerous challenges, enhancing the rational design of heterogeneous catalyst systems to accommodate various types of targeted reactions is of critical importance. In order to achieve accuracy and reliability in research on heterogeneous catalysis, holistic strategies for constructing highly efficient catalytic systems through a deep understanding of molecular-level reaction mechanisms should be implemented. The primary recommended approaches would involve kinetics and mechanistic study, in situ/operando physicochemical analysis and computational modeling utilizing DFT.

We expect that this collection could attract the special interest of readers in continuing and in-depth research on heterogeneous catalysis by means of the excellent platform that is Molecules.

## Data Availability

Not applicable.
